# Supplementary figures and images for: Discovery of aphid-transmitted Rice tiller inhibition virus from native plants through metagenomic sequencing
Source: PLoS Pathog. 2023 Mar 24;19(3):e1011238. doi: 10.1371/journal.ppat.1011238 (PMC10076042; doi:10.1371/journal.ppat.1011238)

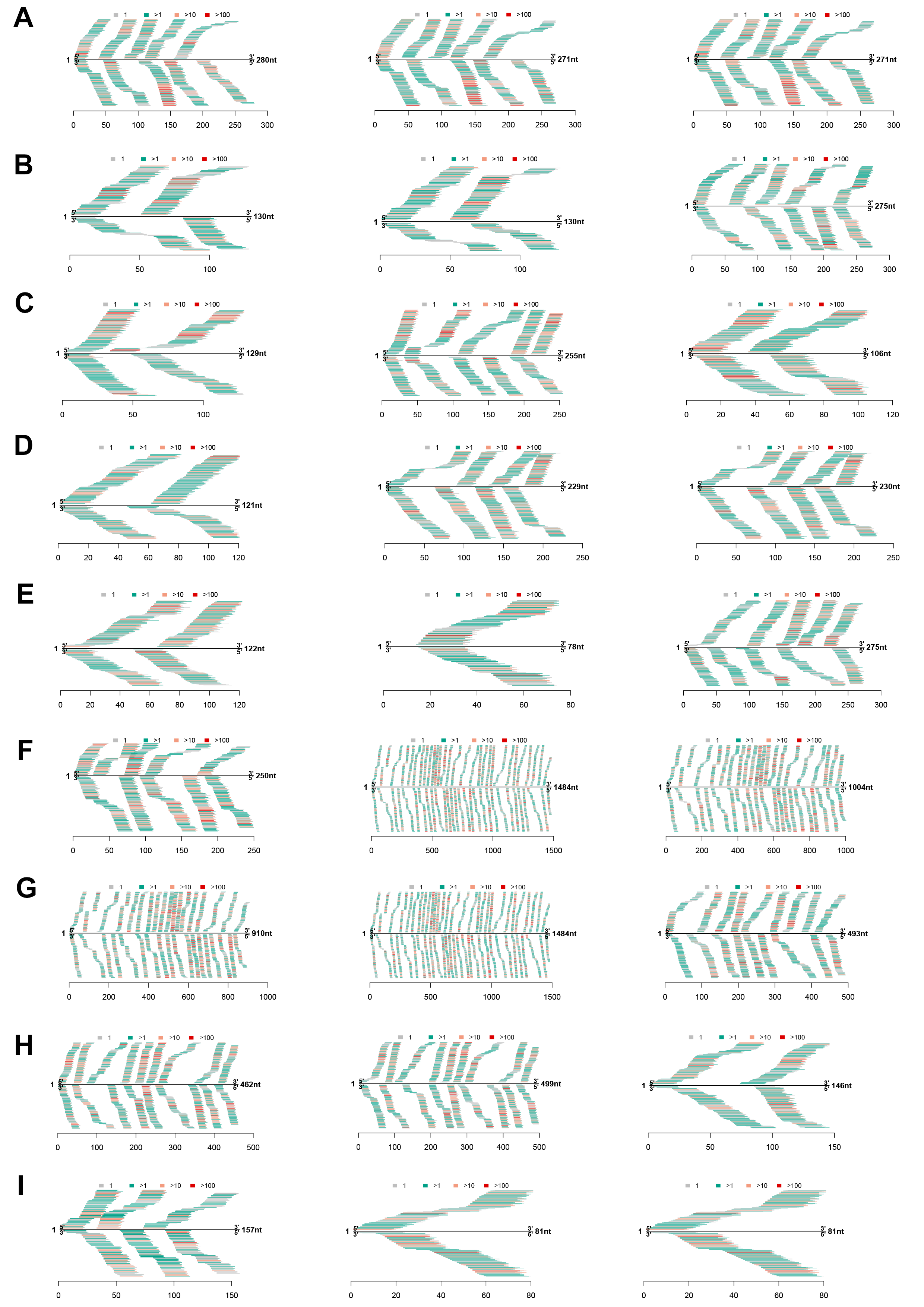

Supplement: S1 Fig — (A-I) Single RTIV contig using vdSAR based on small RNA-seq of wild rice pool. (TIF) [file ppat.1011238.s001.tif]

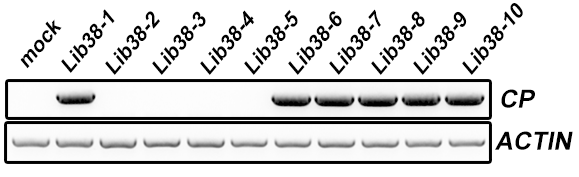

Supplement: S2 Fig — Wild rice individual without RTIV was used as mock. Actin was used as an internal reference. (TIF) [file ppat.1011238.s002.tif]

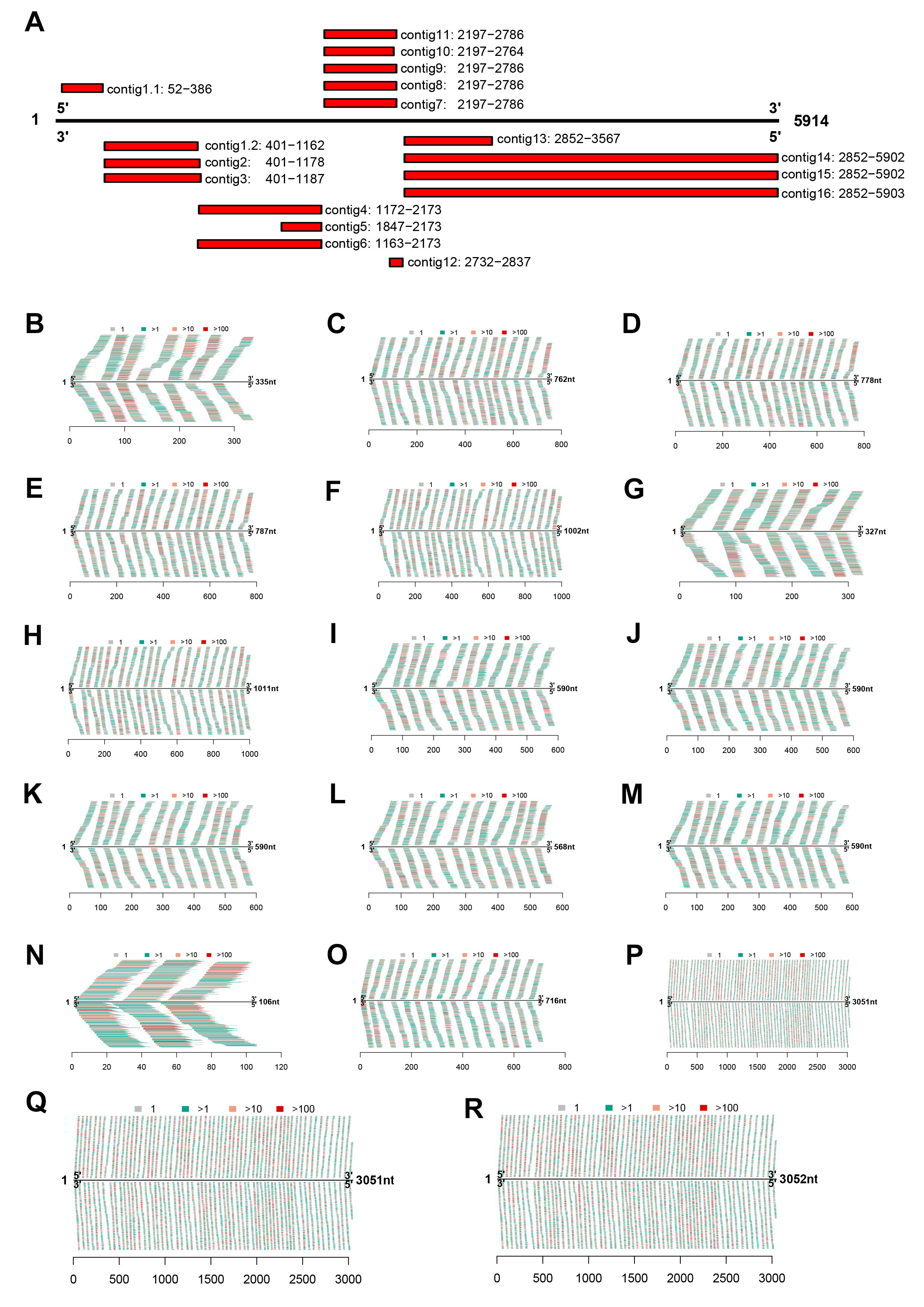

Supplement: S3 Fig — (A-R) Each contig assembled using vdSAR based on small RNA-seq of Colony no.9 plants. (TIF) [file ppat.1011238.s003.tif]

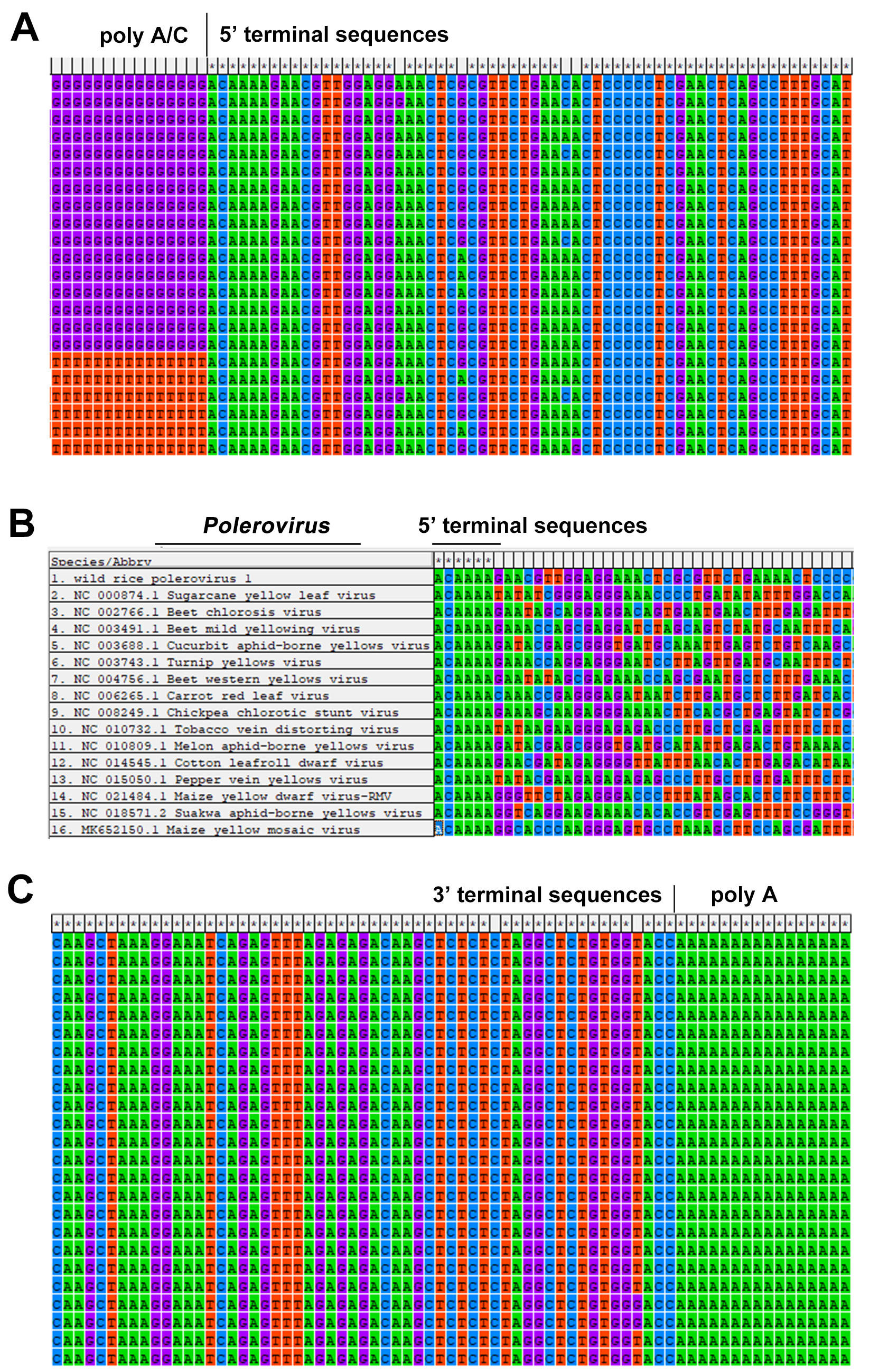

Supplement: S4 Fig — (A) The 5’ terminal sequences were determined by 5’ RACE through adding polyA and polyC. (B) The 5’ terminal conserved sequences in different species of Polerovirus. (C) The 3’ terminal sequences were determined by 3’ RACE through adding polyA. (TIF) [file ppat.1011238.s004.tif]

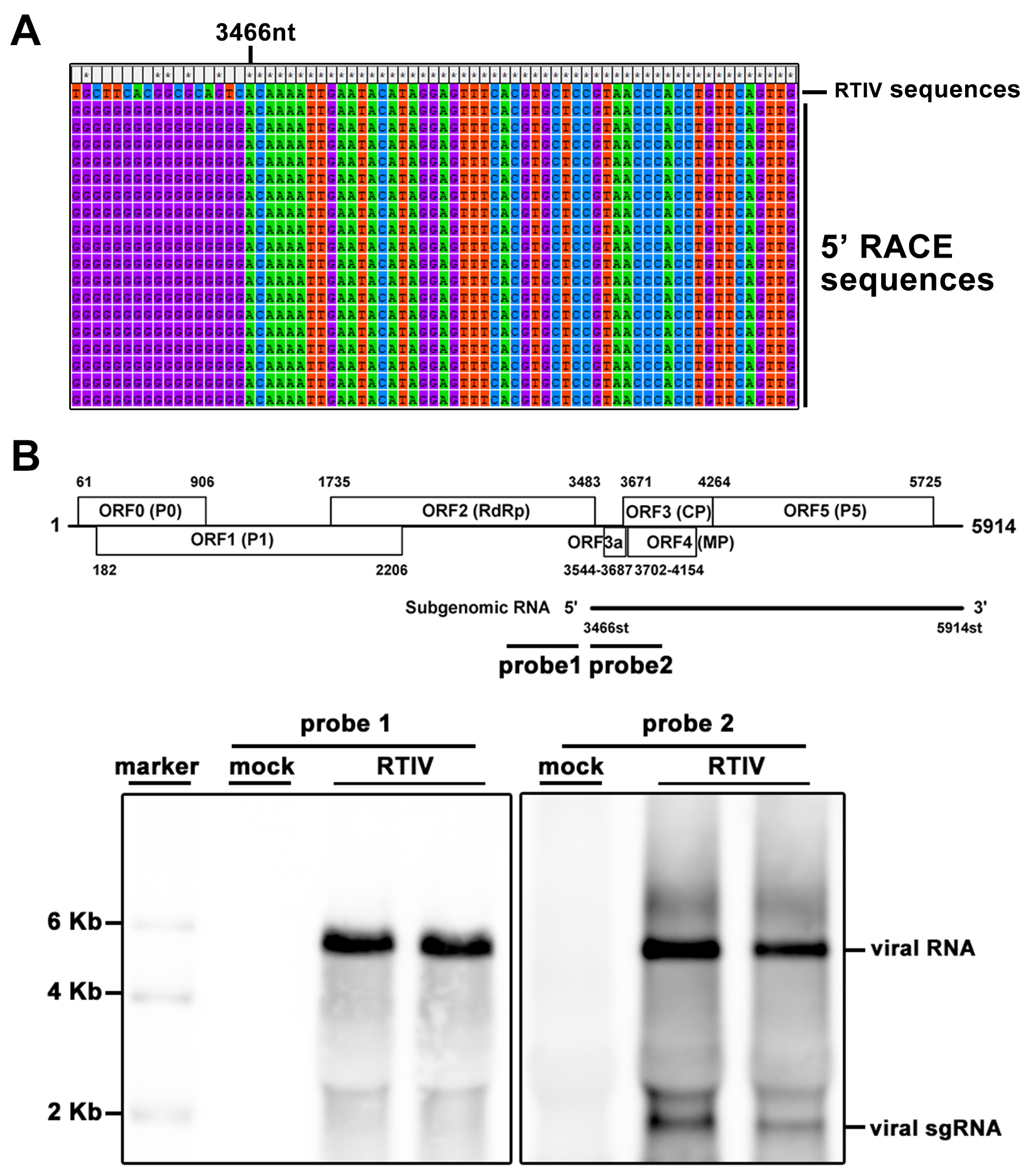

Supplement: S5 Fig — (A) The 5’ terminal sequences of subgenomic RNA were determined by 5’ RACE through adding polyC. (B) Schematic representation of the RTIV genome and subgenomic RNA (up panel), and Northern blot confirmation of the subgenomic RNA in wild rice plants using two different probes shown in up panel (down panel). (TIF) [file ppat.1011238.s005.tif]

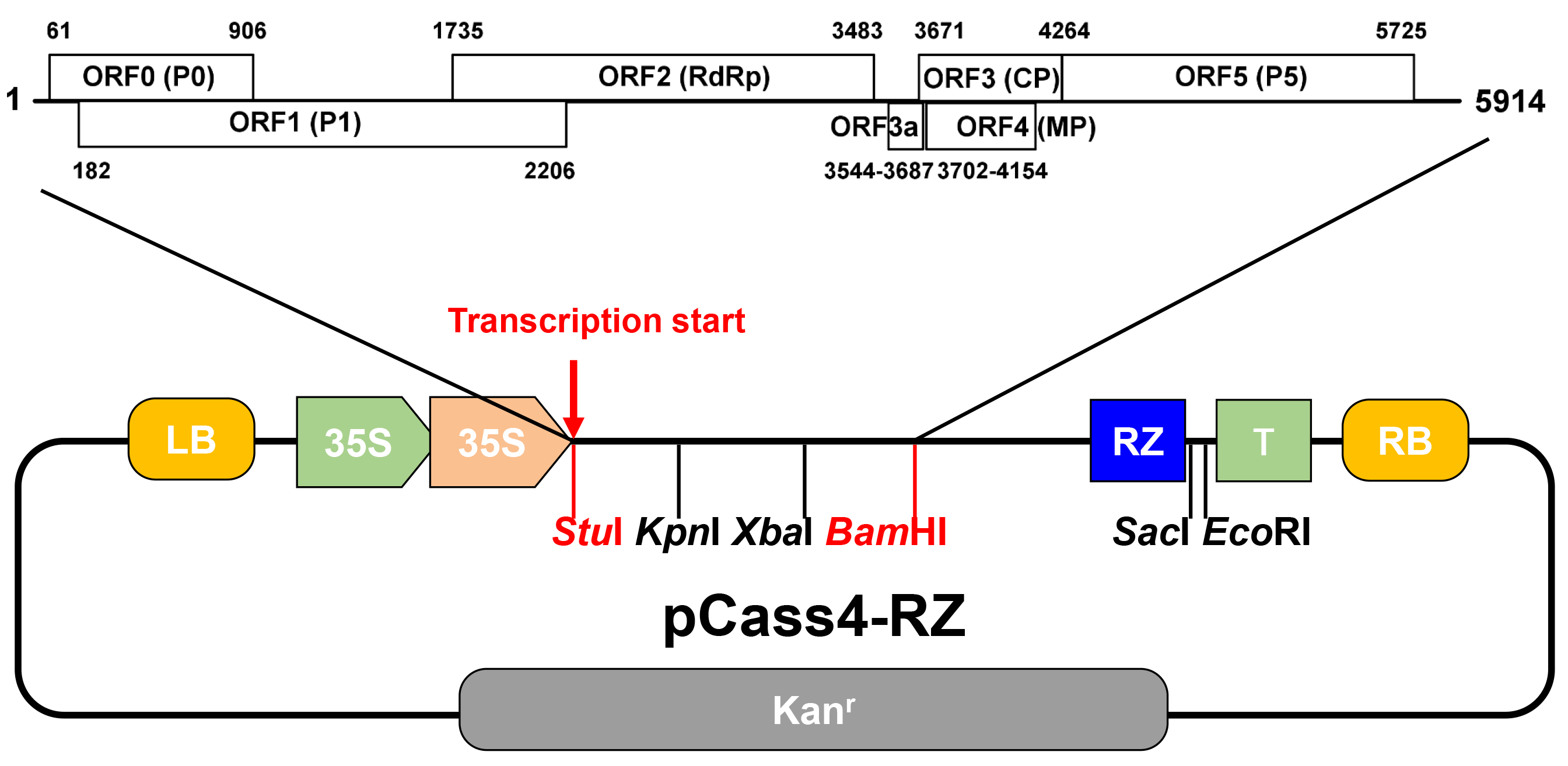

Supplement: S6 Fig — Full-length of RTIV was amplified and ligated to pCass4-RZ vector between Stu I and BamH I sites. Primers used for vector construction listed in S3 Table. (TIF) [file ppat.1011238.s006.tif]

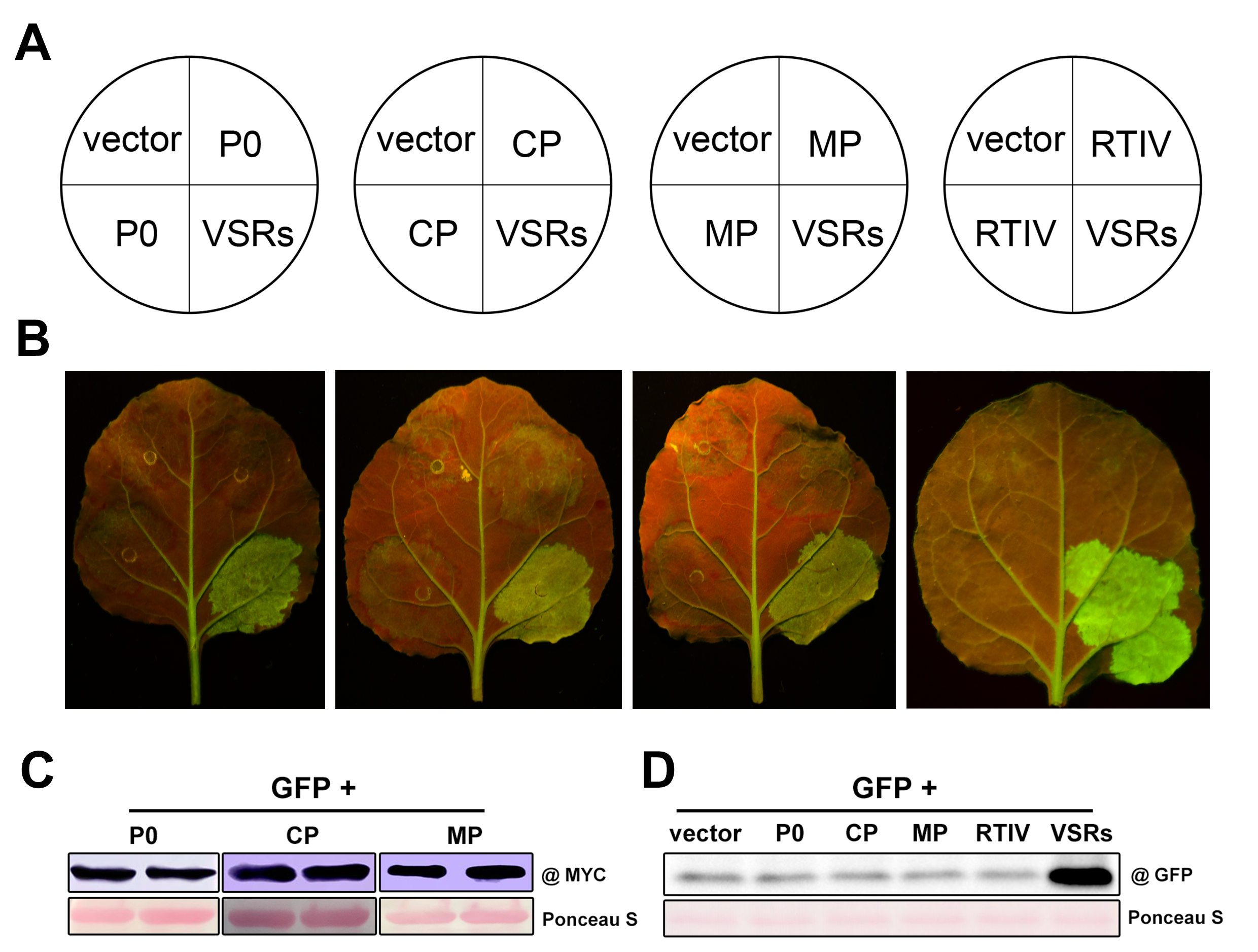

Supplement: S7 Fig — (A) Schematic representation of the Agrobacterium infiltration. (B) 16C transgenic N. benthamiana were respectively co-infiltrated with GFP and P0, CP, MP or full length of RTIV. Empty vector and VSRs were used as negative and positive control, respectively. GFP was visualized under ultraviolet 5pdi. (C) The expression of P0, CP or MP tagged with Myc was confirmed by Western blot, ponceau staining used as loading control. (D) The GFP protein expression levels in different co- infiltrated leaves. (TIF) [file ppat.1011238.s007.tif]

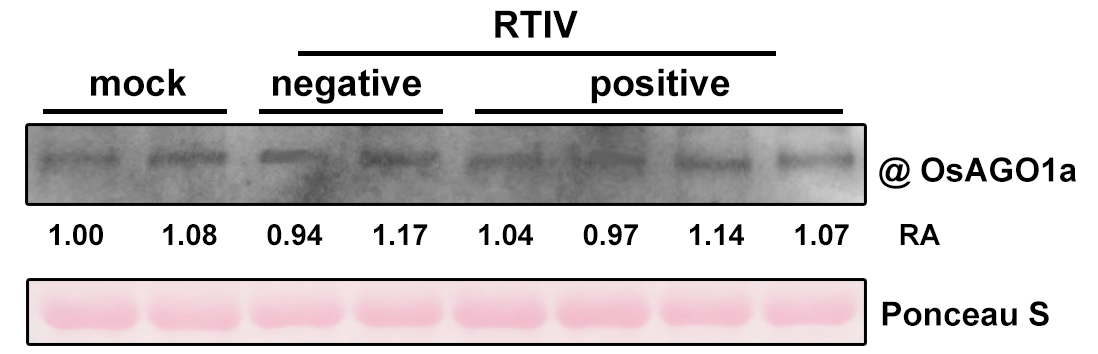

Supplement: S8 Fig — Western blot detection of AGO1a protein accumulation in RTIV-positive and RTIV-negative Zhonghua-11 rice plants at ~30dpi compared to mock Zhonghua-11 rice plants, RA is the relative accumulation of AGO1a normalized to loading control, the same membrane was stained for ponceau to show loading control. (TIF) [file ppat.1011238.s008.tif]

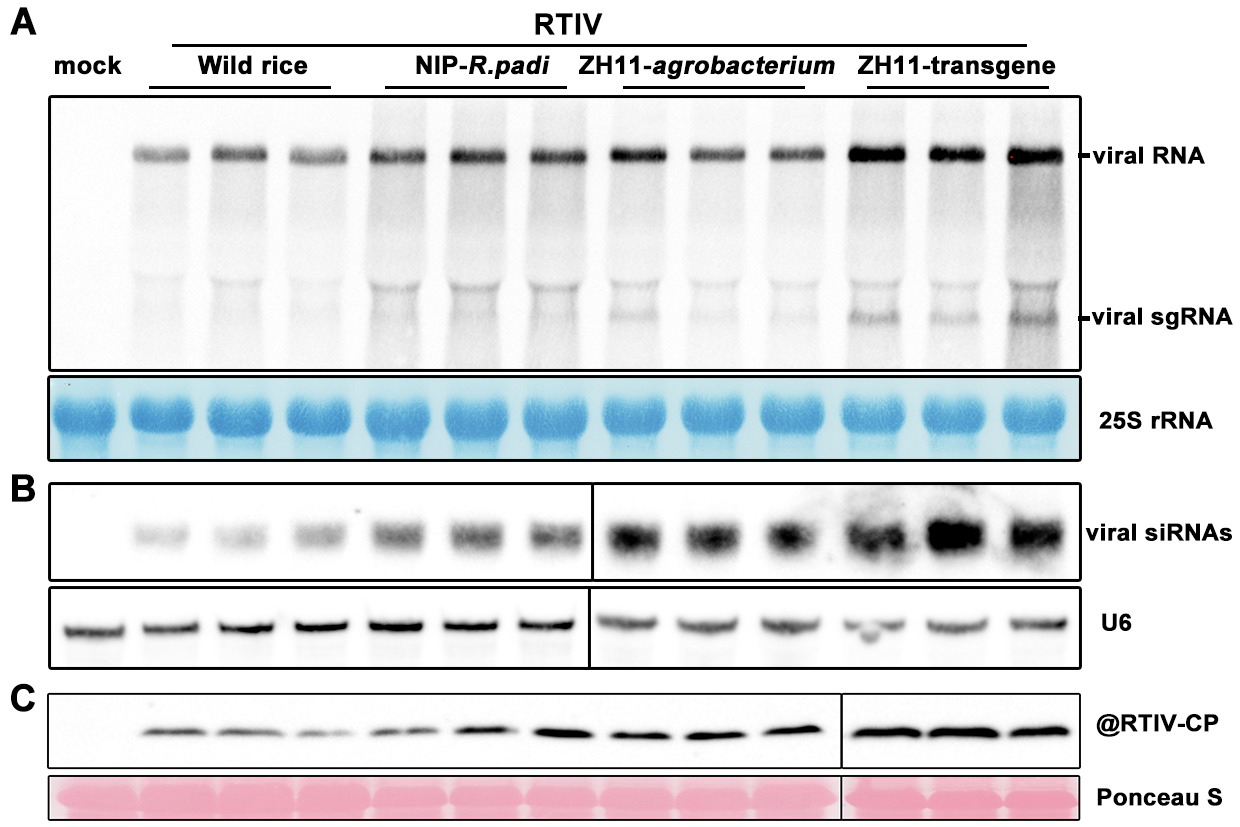

Supplement: S9 Fig — (A) Northern blot detection of viral genomic RNAs accumulation, 25S rRNA in the same membrane was stained to show equal RNAs loading. (B) Northern blot detection of viral siRNAs accumulation, the same membrane was probed for U6 RNA to show equal small RNAs loading. (C) Western blot detection of viral coat protein accumulation, the same membrane was stained for ponceau to show equal protein loading. (TIF) [file ppat.1011238.s009.tif]

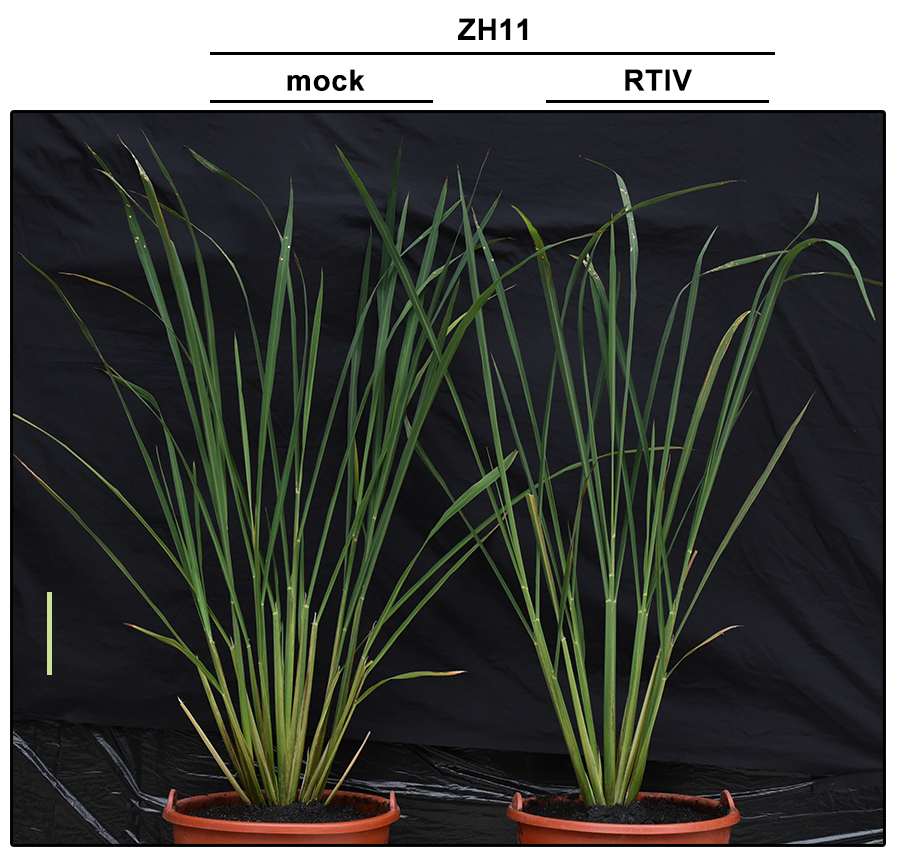

Supplement: S10 Fig — RTIV was acquired from RTIV-positive transgenic rice plants by aphid R. Padi and transmitted to healthy wildtype ZH11. Plants were photographed at ~45 dpi. Scale bar: represent 10 cm. (TIF) [file ppat.1011238.s010.tif]

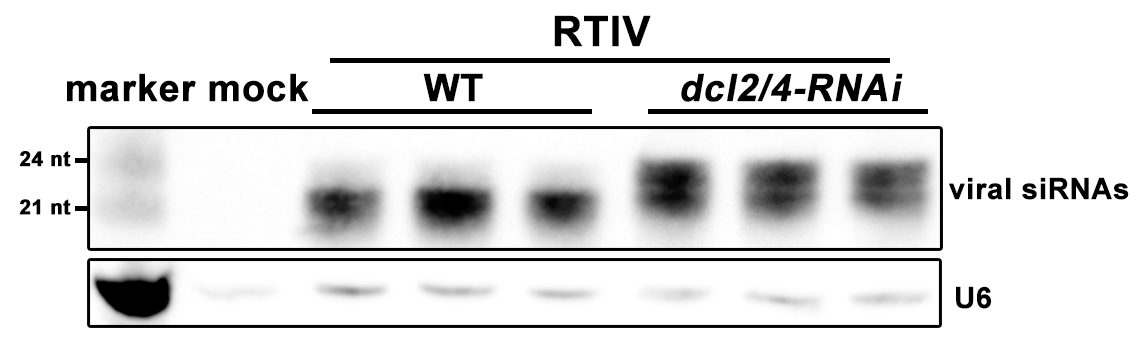

Supplement: S11 Fig — U6 RNA probed in the same membrane to show equal loading, 21 nt and 24 nt siRNA marker were included to show the size of vsiRNAs. (TIF) [file ppat.1011238.s011.tif]
