# Supplementary material for: Discovery of aphid-transmitted Rice tiller inhibition virus from native plants through metagenomic sequencing
Source: PLoS Pathog. 2023 Mar 24;19(3):e1011238. doi: 10.1371/journal.ppat.1011238 (PMC10076042; doi:10.1371/journal.ppat.1011238)
Supplement: S1 Table — Subgenomic region (2449nt from 3466st to 5914st) is red-colored. (DOCX) [file ppat.1011238.s012.docx]

**Table S1. RTIV full-length sequences**

ACAAAAGAACGTTGGAGGAAACTCGCGTTCTGAAAACTCCCCCTCGAACTCAGCCTTTGCATGGAAGTTGTCTCCCTACACACAACAGGTGAAGCACTCTTCCACTTCAAGCAATCAGGCGAGGTTCCGGTTGAACTGGTAGCGGCTGCAGAGTATAACTCAATCGCCACCCACCTAGAGAATGAGCATGGTGAAAACCATGTTATTCGTTTTTACGATTCTCTCATTTCTCTACTGCCAGCCCTGCTACTCCCAGGCTCCTGGGTTGTGGCTCGCCGGGACGAGCCCAGTTGGATATTCATCCGACTGGAATATGTATGCGCTTACACGAGGTGGGCCCTCTCTTGGAATAGGGCCCCAAAATTCCAGGGAGTTACAAAAGACGGGTGTGCCCGTCTACATCAgGGAGTcCAATCAGACAAAGCCACTCTTCATGCATTCCTATGCAGAATTGAGTGCCGCTCTATTTATCAAGATCAAGAGCGATTTCAGGGACTCCTCGAGAAAGGTAGAGCTACATTCACAGCGCTTCTGGATCTCCGTCTCCGAGACGTTGAGAGAAACATTAGTGAATTGTTGGCGTTTCACAGCAGGAATACCATCCGTGCTCCACTCCGGTCTACATCATGGTATTACGGCCACACTGCAACTGATTCAGTTCGTCTTAGCGTTCTGTATTCGATGTGCTATGTGGTTATGTGGCGAACTTTTGACCCATTGGTACGATTTAACTCTAACCGGGTTGCTCGCCGCTGTCGTCAGGGGCATGTACCTAGCCGTGCGTTGGATGTTCTCCGACTGTGCGGTTTTGACATTGCTCCAGCTCCCTTTCTCAATCTGCAGGAAACTTTTCTTGCCAAACCCGATCAAGAAGGAGATGGCGGTCAAGGGACACGTGACTATTAAGATCCGCCAACCCCCCCCAAAGAACTCAGTGCTACAGATTGTTCATCCAGATGACTCTCATGTAGGTTATGGTTCAGCCATATTACTCTGCGATGGAACACCAGCTCTGCTGACCGCAGAACACTGCATTGGGGAGAATCGGGCTGCTCTCGGAGTGAAGACTGGGAATCGCATCCCTCTCGACCAATTCCAAAAGATTCTGGTCTCGAAGAGGTTGGATTTAGCGATCCTCCGAGGTCCACCACAATGGACATCCCTGCTCGGTGTTAAAACCACCAGCTTCGTCAACCACACAAAGGTGACGAAAGGCCCTGTCTCGTGCTACACGATAGATGAAGAGGGCCAGTGGGAAGCAGATTGTGGCAAGATTAACGGCGTTCGAGAAATCGATGGCATCCCTGTCGCCACTGTGCTTTCCAACACAGACGCCGGGTATAGCGGAACCCCTTATTTCAACGGGAAGATTATCCAAGGGGTTCACGTTGGCTGTGATGCAAACCTTAATCACAATCTGATGGCTCTGATTCCATCCATAAAAGGTTTGACTGCCCCGAACTATAAGTTCGAGTCATCTGTCACCGAGGGCAGTATTGTATCCTCAGAAGACTCCTACGTCAAAGCATACGAAGCATATGCCGAGGAACTCTACCAAGAGTACACTCGCTATGGTATGGACGATAGTCGAGCCCGCGATGCAGCGTATGACAACGCTGATTACAATTTTCCAAGGAAATATCGCGAGGAGGGTAGACCCACAGCCCCTGCAGACCTCGTTTCCCGGACTGGACCAACTGCATCCAGTCTTCCCGAGCCTTCTCTTAAACTTCAGGGAAACGAGAAGCGTGGCGCCGACCACGTAGAAAAACCGGCATCCACCCCTATCGTGAGGCCCTCAGATATCAGCGAAACGCTCCGCACAGTTGCAGAAGCGCTGATTGGCCGGGTGGATATCAGGAGAATCGAGGAGATGGTAGTGGAACGACTGTCATCAACCGCGATGAAGAAGCCGAAATCGCCCCCCAGGCGCCGGCGGTCAAAGAAGACATCGAGCAGCGAGCGGCCAAAAACCGTGGACGCGCCGAAAACTACCGCCGCTTCTTCGAATCCAAGTACGACTGGGAAATACGTACCGGTGAAGAAATTCCCGGTTTCTATGAAGTCGGAAGGATCGCGGCCCGCTACTACCCCCCAAAACCACAAGACCTCACGTGGGGGAAGAAACTCATCTCCGACCACCCAGAGCTGGGTGAGAAAGTCGCAGGGTTCGGTTGGCCAGAGTTTGGCCACCAAGCAGAAATGAAATCCCTGCGCCTGCAAGCGGCACGCTGGCTGGAGCGCTCCCAGCCCGCAGAAATCCCAAGTGCCGAGCAGAGAGAGAGCGTGATCCAAAGGACCGTGGAGATGTATAGGGCTGTACATTCGATGAACCCACGGTGCGCCAATGAGAGTTCGCTGAGTTGGAGCGGCTTCTTGAACGATTTCGATGAAGCTGTTCGCTCGCTTGAGCTCTCCGCTGGCGTGGGAATCCCGTACAAAACTCTGGGTGTATCTACCCATCGCGACCTTGTCTGCGACCCAGAGTTTCTCCCCGTTTTGACGCTTCTCGTGTATGATCGTCTATGCAAGTTATCTACACGAGACTGCTCCGGGTTGAGACCTGAAGAACTTGTACGGGAGGGACTGTGCGACCCCATTCGCCTCTTTGTAAAGAAAGAGCCTCATAAACAGTCCAAGCTTGACGAGGGACGCTACCGCCTCATCATGTCTGTCTCCCTAGTGGATCAATTGGTGGCCCGGGTTTTGTTCCAACAGCAAAACAAGACTGAGATCGCTTTGTGGAGAGCGATCCCGAGTAAACCCGGTTTTGGCTTATCCACCGACAGCGAAGCTCTCGAATTTTTCACATCGCTGTCGAGGCTTGTAGGCTGCTCTAACGAGGAGTTGCTCCAAAACTGGAGCACAAAGGTAAGACCTACAGACTGCTCCGGCTTCGACTGGAGCGTCGCTCATTGGATGCTCGAGGATGACATGGAGGTGAGAAACCGCCTCACAATCAATTGTACCGAGCTCACCAGAAGAATGCGCAGTTCCTGGTTGACGTGCCTCAGCAATGCTGTGATCAGCACATCCAATGGCAGCCTTTACGCCCAGAAATTCCCTGGAGTTCAGAAGAGTGGATCATACAATACAAGTTCCTCTAACTCAAGGGTCAGGGTGATGGCCGCTCTTCATTGCGGAGCCACCTGGGCGATAGCCATGGGGGATGATGCGCTGGAGTCCCCCGACTCTGACCTGCGGGGATATAGCAGGTTAGGGTTCAAAGTCGAGGTGTCTGAGAGCCTTGAATTTTGCTCACACATCTTTGAGCAACCAGACCTCGCCAGGCCGGTAAATGTCAACAAAATGATATACCGCTTGATCTTCGGATACAATCCGGCCTGTGGGAGTGAGGAAGTATTGCACAACTACCTCACCGCAGTTGCGTCCGTTCTAATAGAACTGCGCCATTTCCCGCAACTGCTCCCAAAACTCCAACAGTGGCTGCTTCACGGCGCAGTCACAAAATTGAATACATAGGAGTTTCACGTGCTCCGTAACCCACCTGTTCAGTTGGAAGAGTGGGCAGAAAGTCTGGACATACGCAGCCTGGACTATAAACTTTTGGCAGGTGTTTTGATTGGCATTCTTGTTGCAATCCCTGTCGTTATTGTGGCTGCGTACCTGTCATTCCTACGAATCAGCGCCCATATACGCGCGATTGTTAATGAATACGGGCGGTAATCGCGCCAGGAGGAATGTCAGAAGGAGAGCTAACCGTCGTCGGGCAGTTAGGCAGGTGGTCGTGGTCAGGCCTGCCCGAAGAACTCGAAGAGTACGACGACGTGGAGCACTCAGCGGAGGAGGTACTGTGCGAGGACCTGGACGCCGGAGCAATGGGGAAGTTTTCACTTTCACGGTTGACGATCTCAAAGCCAACAGTTCCGGGATCCTCAAATTCGGACCGAGTTTATCACAGTACGCAGCGTTCTCTAATGGATTACTCAAGTCCTTCCACGAGTATAAAATCACTTCAGTTACAGTACAGTATAACTCGTGCTCCTCCGCCACCACTCCAGGTTCAATCTCACTGGAAGTGGATACATCCTGCTCCCAAACAACAACAGGGTCAAAGATCGTTTCTTTCCCCGTCAAGACCAACGCAAGGAAGACATTCCCCTCGACTTACATCAAAGGGAAAGACTTCCTGACTACGACAGCTGACCAGTTTTGGCTGTTGTACAAAGGTAACGGGTCATCTTCAGACCTAGCAGGACAATTTGTCGTCTCCTTTCACTGTCTGTACCAGAATCCAAAATAGGTAGACGACGCTCCCCCAGCACCCACACCCCAACCAACTCCGGCCCCGACCCCTCCGCCACCAAAAGCGAACCAATTCTTTGGCTATGAGGGTGTCCCCTGCTCTGTGGTGAAGACGCGACTGAACTCAGAATTCATTGACGTGTCTCCCCTTTCATACGTACAGCTGTATTTCTGGAAAGATGAGCAGTGGTCCGTCGAGACGTTGTCCGCTGGTTATTACAATAACAACCGAGAGTTCGCCACCCCCTATATCCTGATCCCAGTTGAGAAAGGGGAATACTCCGTCTACATAGAATGTGAGGGCTTTCAAGCTGTGAAGGCCAAAGATGGCTCGAACAAAGGTAAAATGAGCGGCTTCATCTCATATGATGAATCGAGAAGCGGTTGGCAGGTGTATAACTACAGTGGGTGTACTATCACAAAGCTCCAAACGGCCGACTCAAAGGTCCCTGGTCATCCTGATCTTAAAGTAAACGGCTGTGAGTTCACAGATCAACTAGTGGAGCGTGATTTTTATTGTTCCTTTCACCTTGTGGCTGACAAGCAGGCATACTTTGCCCTTCAAGCCCCACCCATAGAGAAATCTGATCATCATAACTATGTGGTGTCCTATGGATCATACACAGAGAAGAGCTTAGAATGGGGCTCATTATCTGTTTCAATGGATGAGGTGAATGAGACGGGAGCGTATTATTCCTCGAAATGGGATAAGAGGAGCCATTTGGCAGCACGACTTGGTAGACAGAAACCCGCCCCTTCAGAAGAGTATGTCGCACCAGCACCAGTCGTTCCGGATAGTCCGACTGTAGCGGAAGCCCGGAATCCTGTAACTCCTCAGGTAAAACAAAAGGAGCCCGAAGTTAGAATATCGGACAAGCCCGACCCACGGGTAATGAGCTGGGTTCATGACCAGCCAGTTGTCCAAGCCGTCTTGGACGGGGAGGCCGAACACGAGATCGTGTATCCCCCAGTCCCACCACCGTTGACGCCCGCTGACACGCGTCGACAAGCTGGAAAGGTGGTTAGAGCTTTGATGGCTAACGCCAGTGAGACGCCAGAACCTCAAGTTGAGCATAGTTCGCGTCCTTCATCACCGGCGATGCAAGTGGAAAGGCCAGCAAGCCCCATAAGAAGTGAGACCCCGACTTTGTCTGGACGATCTATCACAGGTGGCACGCTTCGGCCAAAACTGTCGACAAGTTCGAGGGCTGGTTCGCCCCCTCGGGATGTCTCCCCAACTCGGAGTGAGACTCCCACATTGTCTGGCAGCTCCCTTCGGGGAGGTTCACTGCGCCCAAACCTGGGTGCATCCACTGCACAACTCGGCGAGATGACCCAAGAGGAGAGGAACACTTACAAGCGTATACTCGATTCTCTTGGTTCAACGAAAGCTGCTCAGTATGCAGCCAACATTGGGAAGGCCATCCCAACACCTCGACGCCGAGGCTTCTTCGGGTGAGTCGAGCTCGAGACAATAAACGTAGCCCTTTCTGCCATAGGTGAAGGCAGGCTCGCCTCCGAGCATAACAGAGAGACAGGTTAGCAACCTGGCCGCCCCtTCGGTCGTATCCGACGATCCAAGTGTGGTTTCCAAGCTAAAGGAAATCAGAGTTTAGAGAGACAAGCTCTCTCTAGGCTCTGTGGTACC

Red-colored region: subgenomic RNA (2449nt from 3466^st^ to 5914^st^).
