# Supplementary material for: Discovery of aphid-transmitted Rice tiller inhibition virus from native plants through metagenomic sequencing
Source: PLoS Pathog. 2023 Mar 24;19(3):e1011238. doi: 10.1371/journal.ppat.1011238 (PMC10076042; doi:10.1371/journal.ppat.1011238)
Supplement: S2 Table — Known viruses in Sobemoviridae used for analysis of phylogenetic tree and sequence similarity with RTIV. (DOCX) [file ppat.1011238.s013.docx]

| **Table S2: GenBank Accession Number used in this work** | | |  |
| --- | --- | --- | --- |
| **Family** | **Genus** | **virus** | **GenBank Accession No.** |
| ***Solemoviridae*** | ***polerovirus*** | Maize yellow mosaic virus | MK652150 |
|  |  | Sugarcane yellow leaf virus | NC_000874.1 |
|  |  | Potato leafroll virus | NC_001747.1 |
|  |  | Cereal yellow dwarf virus RPS | NC_002198.2 |
|  |  | Beet chlorosis virus | NC_002766.1 |
|  |  | Beet mild yellowing virus | NC_003491.1 |
|  |  | Cucurbit aphid-borne yellows virus | NC_003688.1 |
|  |  | Turnip yellows virus | NC_003743.1 |
|  |  | Cereal yellow dwarf virus RPV | NC_004751.1 |
|  |  | Beet western yellows virus | NC_004756.1 |
|  |  | Carrot red leaf virus | NC_006265.1 |
|  |  | Chickpea chlorotic stunt virus | NC_008249.1 |
|  |  | Tobacco vein distorting virus | NC_010732.1 |
|  |  | Melon aphid-borne yellows virus | NC_010809.1 |
|  |  | Wheat yellow dwarf virus GPV | NC_012931.1 |
|  |  | Cotton leafroll dwarf virus | NC_014545.1 |
|  |  | Pepper vein yellows virus 1 | NC_015050.1 |
|  |  | Suakwa aphid-borne yellows virus | NC_018571.2 |
|  |  | Maize yellow dwarf virus RMV | NC_021484.1 |
|  |  | Pepo aphid-borne yellows virus | NC_030225.1 |
|  |  | Faba bean polerovirus 1 | NC_055495.1 |
|  |  | Pumpkin polerovirus | NC_055513.1 |
|  |  | Wheat leaf yellowing-associated virus | NC_035451.1 |
|  | ***Enamovirus*** | Pea enation mosaic virus 1 | NC_003629.1 |
|  | ***polemovirus*** | Poinsettia cryptic virus | NC_011543.1 |
|  | ***Sobemovirus*** | Southern bean mosaic | NC_004060.2 |
