# Supplementary material for: Discovery of aphid-transmitted Rice tiller inhibition virus from native plants through metagenomic sequencing
Source: PLoS Pathog. 2023 Mar 24;19(3):e1011238. doi: 10.1371/journal.ppat.1011238 (PMC10076042; doi:10.1371/journal.ppat.1011238)
Supplement: S3 Table — All primers used for different PCR amplifications in this work. (DOCX) [file ppat.1011238.s014.docx]

| **Table S3: Primers used in this work** | |
| --- | --- |
| **Name** | **Sequence (5' to 3')** |
|  | **Primers used for PCR detection of RTIV** |
| RTIV-CP-F3671 | ATGAATACGGGCGGTAATCG |
| RTIV-CP-R4258 | TGGATTCTGGTACAGACAGTG |
| *OsACTIN*-F | CTTCGTCTCGACCTTGCTGGG |
| *OsACTIN*-R | GAGAAACAAGCAGGAGGACGG |
|  |  |
|  | **Primers used for the probe amplification for detecting RTIV** |
| Probe1-RTIV-F2955 | CACAATCAATTGTACCGAGCTCAC |
| Probe1-RTIV-R3465 | CTGACGCGGCACTTCGTCGGTGACAA |
| Probe2-RTIV-F3466 | ACAAAATTGAATACATAGGAGTTTCACG |
| Probe2-RTIV-R4120 | GAATGTCTTCCTTGCGTTGGTC |
|  |  |
|  | **Primers used for PCR amplification of full length RTIV for**  **pCass4-Rz vector construction** |
| pCass4-Rz-*Stu* I-RTIV-F | agttcatttcatttggagaggACAAAAGAACGTTGGAGGAAACTCGCGT |
| pCass4-Rz-*BamH* I-RTIV-R | cggtgacagggtatcggatccGGTACCACAGAGCCTAGAGAGAGCTTGTC |
|  |  |
|  | **Primers used for qRT-PCR analysis of tiller genes transcript levels** |
| CCD8B(D10)-qF | GCTGTACAAGTTCGAGTGGCAC |
| CCD8B(D10)-qR | CGGTGTTGGCGTTGTGCTCG |
| MOC1-qF | GTGTTGTATCAAGATGCCCTTG |
| MOC1-qR | CAAGTCTCCAACCATCTCATG |
| ERG1-qF | TGCAGGCAAGATAGACCCGTAC |
| ERG1-qR | GAAGTCGTCCCGTGAGAAGGT |
| D53-qF | GCGCAAGTGGCATTACAGCTG |
| D53-qR | AGCCGCAGGCAGTACTCATTC |
| OsACTIN-qF | CAGCCACACTGTCCCCATCTA |
| OsACTIN-qR | AGCAAGGTCGAGACGAAGGA |
|  |  |
|  | **Primers used for PCR amplification of genes for vector construction** |
| pCAMBIA1300-P0-F | tctgaagaggacttgaatggtaccATGGAAGTTGTCTCCCTACACAC |
| pCAMBIA1300-P0-R | tacgaacgaaagctctgcaggtcgacTTAATAGTCACGTGTCCCTTGACCG |
| pCAMBIA1300-CP-F | tctgaagaggacttgaatggtaccATGAATACGGGCGGTAATCGC |
| pCAMBIA1300-CP-R | tacgaacgaaagctctgcaggtcgacCTATTTTGGATTCTGGTACAGACAG |
| pCAMBIA1300-MP-F | tctgaagaggacttgaatggtaccATGTCAGAAGGAGAGCTAACCGTC |
| pCAMBIA1300-MP-R | tacgaacgaaagctctgcaggtcgacTCAGGAAGTCTTTCCCTTTGATG |
|  |  |
| F, forward; R, reverse; lowercase, adaptor sequence was added for vector construction | |
